# Supplementary material for: Causal Relationship between Aging and Anorexia Nervosa: A White-Matter-Microstructure-Mediated Mendelian Randomization Analysis
Source: Biomedicines. 2024 Aug 16;12(8):1874. doi: 10.3390/biomedicines12081874 (PMC11351342; doi:10.3390/biomedicines12081874)
Supplement: Supplementary file 1 [file biomedicines-12-01874-s001.zip › Supplementary Figure/Figure S8/Leaveoneout plot of MR analysis for TL to ubm-b-1458.pdf]

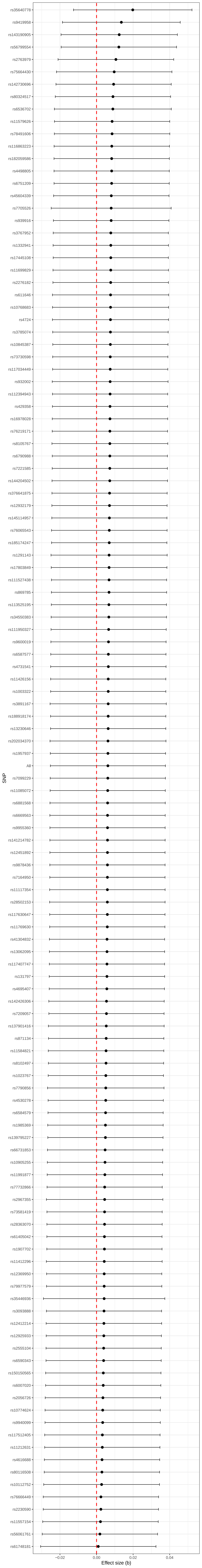
rs35640778
rs9419958
rs143190905
rs56799554
rs2763979
rs75664430
rs142730696
rs80324517
rs6536702
rs11579626
rs78491606
rs116863223
rs182059586
rs4498805
rs6751209
rs45604339
rs7705526
rs939916
rs3767952
rs1332941
rs17445108
rs11699829
rs2276182
rs611646
rs10768683
rs4724
rs3785074
rs10845387
rs73730598
rs117034449
rs932002
rs112394943
rs429358
rs16978028
rs76219171
rs8105767
rs6790988
rs7221585
rs144204502
rs376641875
rs12932179
rs145114957
rs76065543
rs185174247
rs1291143
rs17803849
rs111527438
rs869785
rs113525195
rs34550383
rs111950327
rs9600019
rs6587577
rs4731541
rs11426156
rs1003322
rs3891167
rs188918174
rs13230646
rs202034370
rs1957937
All
rs7099229
rs11085072
rs6881568
rs6669563
rs9955360
rs141214782
rs12451892
rs9878436
rs7164950
rs11117354
rs28502153
rs117630647
rs11769630
rs41304832
rs13062095
rs117407747
rs131797
rs4695407
rs142426306
rs7209057
rs137901416
rs871134
rs11584821
rs8102497
rs1023767
rs7790856
rs4530278
rs6584579
rs1985369
rs139795227
rs66731853
rs10905255
rs11991877
rs77732866
rs2967355
rs73581419
rs28363070
rs61405042
rs1907702
rs11412296
rs12369950
rs79977579
rs35446936
rs3093888
rs12412214
rs12925933
rs2555104
rs6590343
rs150150565
rs6007020
rs2056726
rs10774624
rs9940099
rs117512405
rs11212631
rs4616688
rs80116508
rs10112752
rs76666449
rs2230590
rs11557154
rs56061761
rs61748181
SNP
−0.02
0.00
0.02
0.04
Effect size (b)
